# Imagery Rescripting and Emotional Regulation: Evidence from Neuroimaging Studies

**DOI:** 10.1192/j.eurpsy.2025.749

**Published:** 2025-08-26

**Authors:** M. Ribeiro, E. de Lanna

**Affiliations:** 1 Psychometrics, Brain Matters Research, Delray Beach, United States; 2 Psychology, Universidade Federal Fluminense, Volta Redonda; 3 Psychology, Pontifical Catholic University of Rio de Janeiro, Rio de Janeiro, Brazil

## Abstract

**Introduction:**

Dysfunctions in mental imagery are linked to various psychopathologies, including intrusive memories from trauma, distorted perceptions of reality, and mood disorders. Therapeutic approaches, such as Imagery Rescripting (ImRs), address emotional dysfunctions by modifying mental images. ImRs is a cognitive-behavioral process requiring substantial visuospatial working memory, where distressing memories are recalled and actively altered to reduce their emotional impact. Neuroimaging studies suggest that ImRs engages mechanisms related to sensory perception and autobiographical memory recall.

**Objectives:**

The objective of this paper is to review recent functional neuroimaging evidence on the effects of ImRs on emotional regulation. Specifically, it seeks to explore the neurological mechanisms of ImRs, focusing on its interaction with visuospatial working memory and sensory-perceptual processes to influence emotional outcomes.

**Methods:**

An integrative review of neuroimaging studies on ImRs was conducted using databases like PubMed and Google Scholar, with keywords such as “ImRs” and “functional MRI (fMRI).” The review focused on studies from the last 10 years. Brain areas involved in emotional regulation, such as the visual cortex, amygdala, prefrontal cortex, and hippocampus, were emphasized. Both experimental and clinical studies were included to provide a comprehensive understanding of ImRs’ neurobiological mechanisms.

**Results:**

ImRs improves emotional regulation by activating brain systems involved in visuospatial working memory and emotional responses. Neuroimaging studies reveal that ImRs stimulates the visual cortex and other regions, depending on the type of mental imagery. Emotional systems responsible for primary responses like fear and reward are influenced through these sensory-perceptual processes. Since these emotional systems develop before language, ImRs effectively modifies emotional responses tied to distressing memories by altering the brain’s sensory processing. The findings suggest that ImRs reshapes neural pathways related to visuospatial memory and emotional processing, with significant activation of the visual cortex, amygdala, and prefrontal cortex during rescripting. This highlights its potential as a therapeutic tool for emotional dysfunctions, such as trauma and mood disorders. However, gaps remain in fully understanding its long-term neurological effects and the complete range of brain regions affected by ImRs.

**Conclusions:**

This review highlights the potential of ImRs in regulating emotions by influencing key brain regions. While ImRs shows promise in modifying emotional responses and neural pathways, there are still gaps in understanding its long-term effects. Future research should focus on these gaps, using advanced neuroimaging techniques and examining its effects across diverse clinical populations to fully elucidate its neurobiological mechanisms and optimize therapeutic applications.

**Disclosure of Interest:**

None Declared

